# Effect of microtopography on osseointegration of implantable biomaterials and its modification strategies

**DOI:** 10.3389/fbioe.2022.981062

**Published:** 2022-09-26

**Authors:** Yingying Zhang, Zhenmin Fan, Yanghui Xing, Shaowei Jia, Zhongjun Mo, He Gong

**Affiliations:** ^1^ Beijing Key Laboratory of Rehabilitation Technical Aids for Old-Age Disability and Key Laboratory of Human Motion Analysis and Rehabilitation Technology of the Ministry of Civil Affairs, National Research Center for Rehabilitation Technical Aids, Beijing, China; ^2^ School of Mechanical Engineering, Jiangsu University of Technology, Changzhou, China; ^3^ Department of Biomedical Engineering, Shantou University, Shantou, China; ^4^ School of Biological Science and Medical Engineering, Beihang University, Beijing, China

**Keywords:** implants, microtopography, surface modification, osseointegration, mechanism

## Abstract

Orthopedic implants are widely used for the treatment of bone defects caused by injury, infection, tumor and congenital diseases. However, poor osseointegration and implant failures still occur frequently due to the lack of direct contact between the implant and the bone. In order to improve the biointegration of implants with the host bone, surface modification is of particular interest and requirement in the development of implant materials. Implant surfaces that mimic the inherent surface roughness and hydrophilicity of native bone have been shown to provide osteogenic cells with topographic cues to promote tissue regeneration and new bone formation. A growing number of studies have shown that cell attachment, proliferation and differentiation are sensitive to these implant surface microtopography. This review is to provide a summary of the latest science of surface modified bone implants, focusing on how surface microtopography modulates osteoblast differentiation *in vitro* and osseointegration *in vivo*, signaling pathways in the process and types of surface modifications. The aim is to systematically provide comprehensive reference information for better fabrication of orthopedic implants.

## 1 Introduction

The replacement and healing of bone tissue has become a major challenge worldwide, due to the high incidence of accidents and the prevalence of age-related diseases. Application of bone implant is one of the effective means for the treatment of traumatic and congenital bone defects. However, there is usually no direct contact between the implant and the host bone, which leads to poor osseointegration and implant failure (Xia et al., 2018). Poor interfacial bonding between host bone and implant was mainly associated with minimal osseointegration leading to implant instability, micro-movements and fibrous tissue formation (Srivas et al., 2019). Osteointegration relies not only on mechanical interdigitation to ensure initial fixation, but also on cellular interactions at the surface level to promote osteoconduction, osteoinduction, and healing during the early stage of implantation.

**FIGURE 1:**
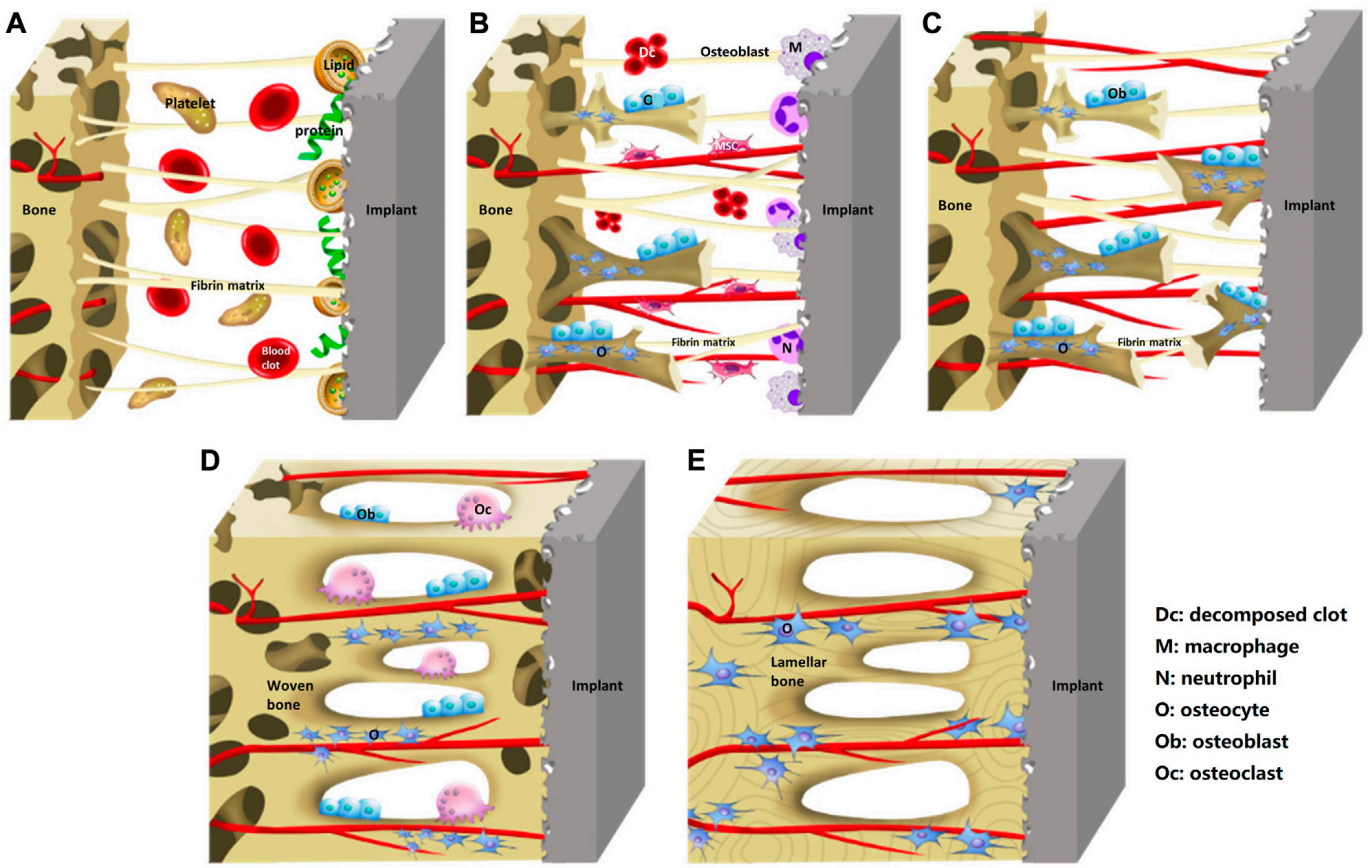
Schematic diagram of the process of implants osseointegration. **(A)** Blood clot and fibrin matrix formation. **(B)** Woven bone formation. **(C)** Distance osteogenesis and contact osteogenesis. **(D)** Newborn woven bones fill up the gap. **(E)** Woven bone matures into lamellar bone. Reprinted with permission (Liu et al., 2020a). Copyright 2019, the Authors. Published by Wiley.

Osseointegration is a dynamic process involving a sequence of cascade responses in which the surface properties of implant play a crucial role (Figure 1, Liu et al., 2020a). Once the implant is placed into the body, an inflammatory response will occur and lead to the release of various proteins such as growth factors and cytokines that form a blood clot (Lotz et al., 2018). The proteins will soon be absorbed by the implant surface from the blood clot, which may act as a signal for cell migration and proliferation (Rivera-Chacon et al., 2013). The specific types of proteins and firmness of adhesion depend largely on the characteristics of the implant surface, such as roughness, and hydrophilicity (Boyan et al., 2017). Cytokines and growth factors stimulate the recruitment of mesenchymal stem cells (MSCs), which proliferate, then differentiate into osteoblasts that are responsible for producing a mineralized matrix and immature woven bone surrounding the implant. Over time, the woven bone gradually matures into lamellar bone, further reinforcing the bone-implant interface (Popat et al., 2007). However, MSCs can also differentiate into fibroblasts that may stimulate the formation of a fibrous membrane on the implant surface and impede the process of bone ingrowth (Razzouk and Schoor, 2012). It is influenced by the properties of implant surface. Therefore, surface properties play a crucial role in the long-term stability and functionality of implants.

An ideal implant should have the ability to stimulate osteogenic cells responses such as adhesion and proliferation on the surface. The interface between implants and the surrounding tissue is the critical area and plays a pivotal role in directing cell responses to biomedical devices (Lumetti et al., 2016). Implants surface have effects on cellular responses including contact guidance and influence on cellular functions. It is widely believed that microstructural surface is beneficial to increase bone-implant contact and anchorage for improving the interface bonding strength (Kubo et al., 2009; Lee et al., 2015; Zhu et al., 2018; Piglionico et al., 2020; Di Tinco et al., 2021). Therefore, surface modification is necessary and of particular interest to improve implants bioactivity and other biofunctionalities, and hence enhances the cellular and tissue responses (Lu et al., 2020; Wang et al., 2020; Xue et al., 2020). In an attempt to improve bone formation and implant fixation, implant surface with various microtopographical cues have been investigated to induce the osteogenic differentiation of cells, which may assist more rapid and stable osseointegration, and improve bone bonding at implant surfaces (Karazisis et al., 2016). More attention was paid to the mechanism of interface microenvironment affecting osteogenic behavior in recent years. Although microtopography can enhance osteogenic differentiation of cells, there is no consensus on the optimal structure and feature, and the mechanism is still unclear.

The aim of this review is to provide an overview of the recent experimental evidence to the relationship between microtopography (including microporous structure, microgrooves, micropillars, micropits, nanotubes, and other features at macro-, micro-, and nanoscales) and cell adhesive/osteoinductive properties. Implant with such potential would accelerate implant osseointegration and healing. To this end, this review particularly focuses on surface microtopography modification and its application in implant biomaterials. Briefly, the manuscript has the following aspects: the physical properties of the microtopography, the relationship between the surface microtopography and the cellular *in vitro* and osteogenesis *in vivo*, the potential mechanism regarding how microtopography affects osteogenic differentiation of cells, the presently available strategies of surface microtopography modification and the pros and cons for every technique. It will provide reference for the future design of implant surface morphology.

## 2 Physical properties of microtopographies

Roughness and hydrophilicity are important physical properties of the implant’s microtopography, which have important effects on the biocompatibility. Surface with microstructure creates higher roughness and specific surface area compared to smooth ones, which enhances the contact area between bone tissue and implant; make it more conducive to the anchoring of bone tissue on material, thus improving the stability of bone tissue. Cell adhesion state (Yang et al., 2016), migration (Martínez-Calderon et al., 2016), proliferation (Rabel et al., 2020) and differentiation (Manivasagam and Popat, 2021) can be governed by surface properties.

### 2.1 Surface roughness

Surface roughness of the implant is essential for integration in tissue regeneration (Matos, 2021). Increasing the average roughness of the implant surface was capable of achieving higher rates of osseointegration. This may be due to the positive influence of surface roughness on protein adsorption and osteoblastic function, as well as higher micromechanical retention of bone on rougher substrates. Surface roughness has high surface energy, which enhances initial protein adsorption and facilitates cell interaction at the implant interface (Srivas et al., 2019). Surface roughness can strongly influence cell adhesion (Majhy et al., 2021), migration and the geometric shape of cells (Petrini et al., 2021). Study has shown that higher levels of cell adhesion were obtained on the rougher surfaces functionalized with the peptide. It has been reported that surface roughness affects cell behavior directly by enhancing the formation of focal contacts or indirectly *via* selective adsorption of serum proteins required for cell adhesion (Zhang et al., 2016). By sensing the roughness gradient, the cytoskeleton of cells exhibited higher tension on the rougher surface, which was further transferred to the nucleus and ultimately affected the expression of osteogenic related proteins (Chen et al., 2022).

### 2.2 Surface hydrophilicity

In the presence of the microstructure, the hydrophilicity increased appreciably. The surface wettability can be tuned from hydrophobicity to hydrophilicity by controlling surface topography (Han et al., 2021). The effect of microtopography on surface hydrophilicity has been reported. Compared with untreated surfaces, the contact angle of the microgroove surface is lower (Wang C. et al., 2019). Surface hydrophilicity can modulate the bonding strength, total amount, and conformation of adsorbed proteins that can influence early cell adhesion, proliferation and differentiation. It was found that cells on hydrophilic surface expressed higher level of integrin genes than those on hydrophobic surfaces (Zan et al., 2020). As we know, integrins are a large family of cell surface receptors, which mediate the interactions between cells and matrix (Siebers et al., 2005). They can regulate some members of the cyclin family in a cooperative manner to progress in the cell cycle. The establishment of specific integrin-matrix stimulation can also lead to enhancement gene expression related to differentiation. Studies have demonstrated that integrin mediated focal adhesion maturation promotes osteoblast differentiation (Hendesi et al., 2015). Integrins play an important role in regulating cell behaviors during bone development and repair (Allori et al., 2008). Surface hydrophilicity has been shown to influence osteogenic differentiation of cells *via* integrin (Kwon and Park, 2018). The higher the hydrophilicity of the material, the better the adhesion of the cells and the better the binding ability to the bone (Sun et al., 2021).

## 3 Microtopography on bone formation and osseointegration

An ideal bone implant should promote early cells adhesion, osteogenic differentiation and adequate bone integration at the bone-implant interface. After the material is implanted, osteoprogenitor cells migrate to the implant site and differentiate into osteoblasts. Therefore, the properties of implant surface play critical roles in the interactions between the biological environment and the implant, interacting with cells to harmonize their adhesion, migration, proliferation, differentiation, and the consequential bone formation (Srivas et al., 2019). Various functional microstructure surfaces have been proposed to improve the osteogenic differentiation behavior of osteoprogenitor cells and improve osseointegration, achieving immediate or early functional loading in patients with reduced bone density (Chen et al., 2022).

### 3.1 Osteogenic differentiation behavior of cells

**FIGURE 2:**
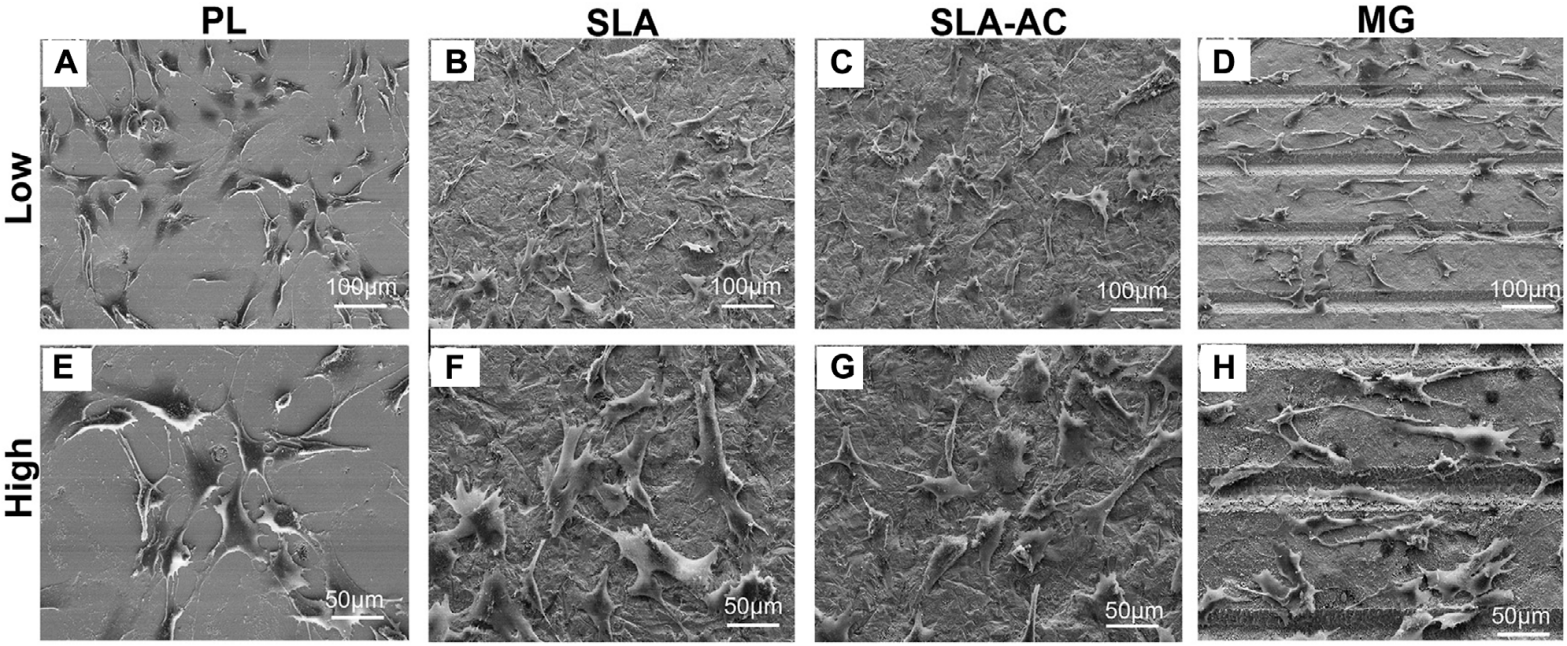
Cell adhesion on materials with different microtopography. **(A–D)** Low magnification and **(E–H)** high magnification. PL: polished group, SLA: sandblasted group, SLA-AC: sandblasted acid-etched group, MG: microgrooves (Sun et al., 2021). Copyright 2021, the Authors. Published by Elsevier.

A number of *in vitro* studies have been performed to evaluate whether surface microtopography can promote cellular reactions that promote bone formation. The most important finding is that textured substrate surfaces can be exploited in inducing osteogenic differentiation of MSCs without any differentiation supplements (Hasturk et al., 2019). At the implant-bone interface, cell adhesion is considered important in defining cells to osteoblast differentiation (Kurashina et al., 2019). What’s important for bone formation is not the number of adhesions that a cell can form, but the size of adhesions (Mas-Moruno et al., 2019). It was reported that stem cells with large spreading and strong contractility prefer osteogenic differentiation, while cells with small spreading and low contractility tend to adipogenic differentiation (Chen and Kawazoe, 2020). Microtopographies with appropriate size and spatial arrangement may provide the basic physical clues required by cell receptors to regulate cell morphology, reorganize cytoskeleton and transmit mechanical signals to the nucleus, which may eventually promote cell differentiation. Numerous studies proved that surface microtopography was able to trigger osteogenic commitment of stem cells, as confirmed by the activation of bone-related markers RUNX2, ALP or ongoing deposition of extracellular matrix supported by the expression of COLI, OPN and OCN (Di Tinco et al., 2020; Xia et al., 2020). For instance, hMSCs on modified topography displayed a noteworthy higher expression of osteogenic transcription factors and the formation of mineralized extracellular matrix when compared to the unmodified materials (Carvalho et al., 2018). Further research also found that the feature size, such as width, height, depth, length, diameter or gap size, can also affect the expression of diverse genes including cellular adhesion, migration and osteogenic differentiation (Stanciuc et al., 2018; Hasturk et al., 2019; Zhu et al., 2019). Therefore, the specific microtopographies feature could induce the osteogenic differentiation of cells, as well as regulate the cell adhesion and morphology (Figure 2, Sun et al., 2021).

### 3.2 Osseointegration *in vivo*

One of the requirements for biocompatibility of implants is that the material should integrate with the bone, known as osseointegration. For a long-term and reliable fixation of implants, osseointegration has been proven to be a powerful solution. Numerous reports have demonstrated that the surface microtopography of implants affects the rate of osseointegration and biomechanical fixation. Rough surfaces seem to accelerate and enhance osseointegration by increasing contact osteogenesis (Pellegrini et al., 2018). Wang and his collaborators (Wang G. et al., 2019) fabricated microgroove with graphene oxide coating on Ti6Al4V alloys implant surface by laser processing and chemical assembly techniques. Results indicated that the binding capacity between bone and implants was obviously enhanced *in vivo*. Wang et al. (Wang G. et al., 2016) constructed hierarchical structure on titanium surface by 6-h treatment of thermal oxidation. Results confirmed that the *in vivo* bone-implant contact showed enhanced osseointegration than the control group. Another study showed that the hierarchical micro-nano (HMN) surface structure remarkably improved the hydrophilicity of Ti6Al4V and the bone-to-implant contact area and new bone volume were significantly improved on the HMN surface structure compared with the mciro-roughened surface on Ti6Al4V (Moon et al., 2017). A recent experiment also showed that micro/nano structure on composite implant could enhance osteogenesis and osseointegration, evidenced with greater bone-implant contacts and push-out force (Wu et al., 2020). Topographical differences can result in differences in bone-to-implant contact and pullout strength *in vivo* (Schwartz et al., 2008). Nanostructuring on a surface with micro-sized roughness has been proposed to induce fast regeneration of the surrounding tissues by regulating the protein interactions (Mendonca et al., 2008). The structural similarity of an HMN structure with native bone that composed of macro-, micro-, and nano-scale components might endow implant surface with favorable osseointegrative activity.

## 4 Signaling pathways of interface morphology on osteogenesis

**FIGURE 3:**
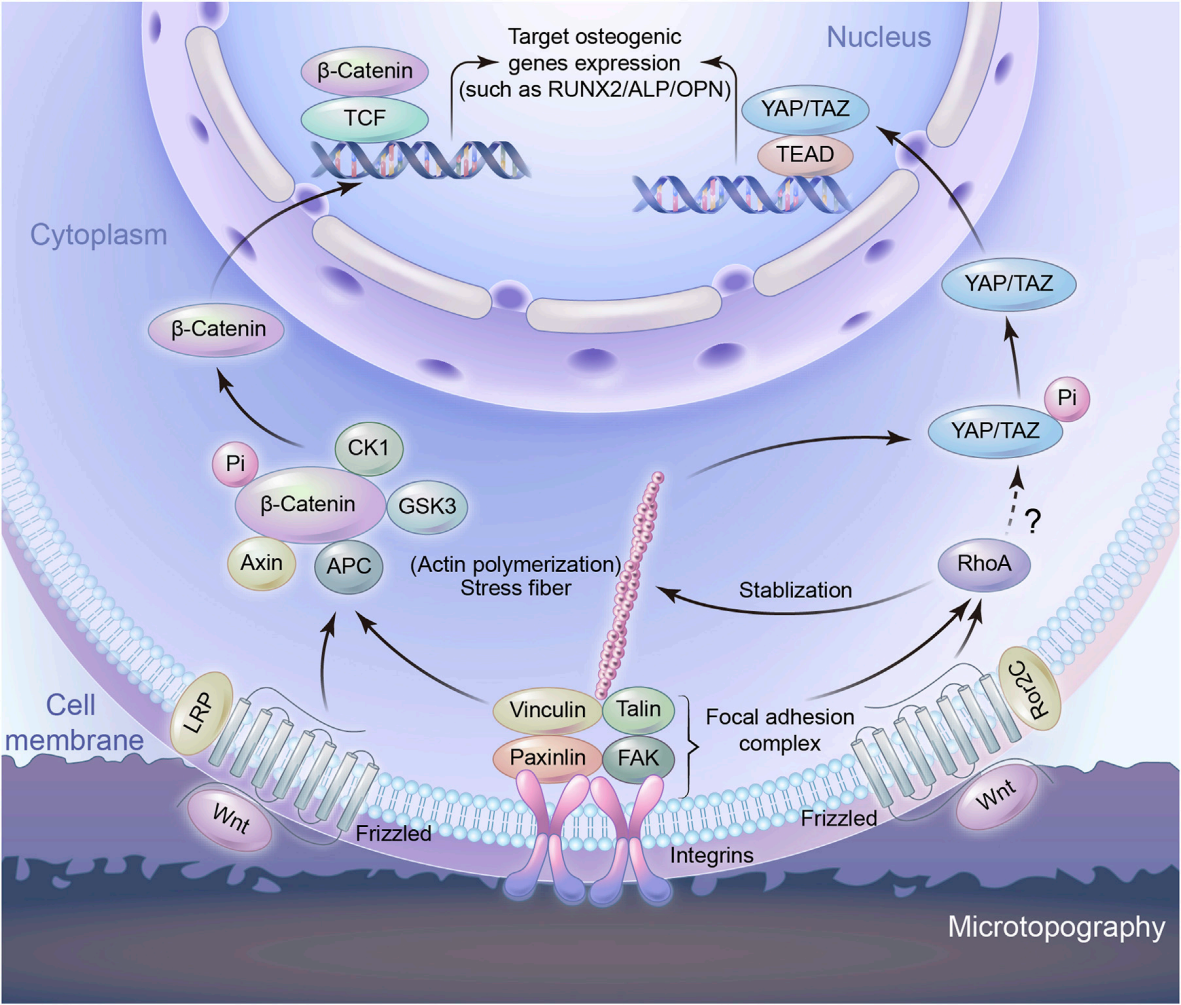
Schematic representation of signaling pathways to regulate osteogenic gene expression induced by microtopography.

Extracellular mechanical signals induced by microtopography are transformed into intracellularly biochemical signals, which are achieved by mechanotransduction process (Burridge et al., 2019; Stewart et al., 2020). This process involves mechanosensing, which in turn activates multiple signaling pathways. Studies have proven that mechanotransduction is involved in micromorphology induced osteogenesis (Fu et al., 2020).Microtopography-induced signals can be transported *via* cytoskeleton and the changes in cell shape to adapt to the underlying surface. Actin cytoskeleton has been shown to remodel in response to microtopography. This remodeling instigates subsequent mechanotransductive pathways, ultimately leading to the osteogenic differentiation (Chen et al., 2022). The molecular mechanisms are mainly known to include Wnt/β-catenin and yes-associated protein and transcriptional coactivator with the PDZ-binding motif (YAP/TAZ) pathway (Figure 3).

### 4.1 Wnt/β-catenin pathway

Wnt signaling is one of the pivotal pathways controlling cell proliferation and differentiation in tissue homeostasis and is known to play a crucial role in bone regeneration, bone formation and osseointegration (Steinhart and Angers, 2018; Staehlke et al., 2020). Hydrophilic rough surface enhanced the expression level of osteogenesis related genes and significantly increased the expression of several Wnt ligands such as Wnt3a, Wnt5a, and the Wnt receptors of the Frizzled family in Wnt signaling pathway (Galli et al., 2010; Donos et al., 2011; Li et al., 2017). Wnt proteins interact with frizzled-LRP surface receptors such that the down stream effector *β*-catenin is increasingly translocated to the nucleus (Li et al., 2017), while at the same time inhibiting the complex that degrades *β*-catenin, namely AXIN1 (Hutchings et al., 2020; Rougerie et al., 2021). Under these conditions, the increased signaling through Wnt/β-catenin signaling pathway is associated with the expression of calcification and osteogenic markers. In addition, RhoA plays a role in controlling Wnt upregulation on microstructured titanium surfaces (Lumetti et al., 2014). Noncanonical Wnt factors bind to receptor Frizzled-Ror2C, which activate RhoA. RhoA, in turns, activates downstream target to phosphorylate Myosin II. Acto-Myosin filaments create cell tension, which facilitates the activation of canonical Wnt/β-catenin signaling. A feedback loop from tension-activated structures controls RhoA activation levels. Actin microfilaments can also control RhoA, too (Lumetti et al., 2014). It is likely that the modification of integrin-based adhesions by the topography is involved at the starting point of the Wnt-mediated response to topography. The silencing of the integrin β3 not only blocked the osteogenic behaviour of MSCs cultured on nanoroughness, but also decreased the expression of *β*-catenin and Frizzled (Lopes et al., 2019). Osteogenic rough surfaces also increase the expression of Wnt5a together with the expression of the integrins in MSC (Olivares-Navarrete et al., 2011). Importantly, the exogenous addition of Wnt5a and Wnt5a knock-down experiments demonstrated that the integrin upregulation was Wnt-dependent (Olivares-Navarrete et al., 2011), suggesting a mutually reinforcing crosstalk between integrin adhesions and Wnt signalling (Barlian and Vanya, 2022). Integrin regulates differentiation through the Wnt pathway, and Wnt increases the expression of integrin, resulting in further regulation of osteogenic differentiation (Sun et al., 2018; Rougerie et al., 2021).

### 4.2 YAP/TAZ pathway

YAP and TAZ are homologous transcriptional coactivators in the Hippo signaling pathway, identified as an important regulatory pathway that restricts cell proliferation and differentiation. Phosphorylated forms of YAP/TAZ are sequestered in the cytoplasm. Both of them act as the nuclear relays of mechanical signals exerted by ECM properties and cell shape and their function depends on Rho and tension of the actin cytoskeleton, as well as cell spreading (Dupont et al., 2011). Studies have shown cell adhesion to matrix proteins can trigger YAP nuclear localization through and integrin/FAK/Src axis, which was involved in nanotube induced differentiation (Tong et al., 2020). Cell mechanotransduction comprises signaling pathways, such as the Rho/ROCK pathway, that result in cell contractility, cytoskeletal and ECM remodeling, or activation of YAP/TAZ that triggers the initiation of a cascade of transcription factors. Contractile actomyosin complexes act as central mediators between microtopography cues and YAP/TAZ signaling in various mechanotransduction environments (Jo et al., 2020). Controlling cells adhesive area on micropatterned substrates can regulate the nucleo-cytoplasmic localization of YAP/TAZ, whose target genes partly explained the proliferation and differentiation of MSCs. Cells with large spreading area exhibited enhanced formation of actin stress fibers and YAP nuclear location. Inhibiting either the actin polymerization or actomyosin contractility reduced the nuclear localization of YAP through disrupting the formation of actin stress fibers. It demonstrates that the mechanical stimuli induced nuclear translocation of YAP/TAZ is determined by cytoskeletal interaction (Wada et al., 2011). A stiffer or tailored surface will increase integrin clustering and FA formation, thereby increasing F-actin polymerization and stress fiber formation. Larger cell spreading area on the microstructure surface will support the translocation of YAP to the nucleus, as well as the expression of preosteogenic genes (Zhang et al., 2016). It is this balance between the YAP/TAZ ratio in cytoplasm and nucleus that plays a role in the regulation of cell differentiation (Heng et al., 2020; Seong et al., 2020; Zhang et al., 2021). The role of TAZ protein in osteogenesis is coactivating transcription by RUNX2 for osteogenesis, while at the same time inhibiting transcription by PPARγ for adipogenesis (Heng et al., 2020).

The Wnt/β-catenin and YAP/TAZ pathways have in common with the remodeling of integrin-mediated adhesions in contact with microtopography. This indicates that the transduction of the topographical cues starts at the cell-implant interface and may be attributed to the features of adhesion sites. Compared to a flat surface, the microtopography provides fewer adhesion sites for cells. Therefore, for cells in order to adhere to the morphology surface steadily, the focal adhesion complex begins to assemble and mature, the F-actin becomes strong and stable. F-actin is one of the major components of the cytoskeleton and can be regulated by RhoA (Tong et al., 2020). Integrins couple the substrate to the other focal adhesion proteins so as to facilitate cell attachment. The adhesion mediated topography sensing involves the signaling pathways classically associated with osteogenic differentiation. Topography, however, can be sensed by the cell as a whole due to the morphological deformation it imposes. Deformation induced microtopography sensing is still unclear, because it involves the whole cell as a unique and complex mechanical unit. Therefore, that is really an attractive future challenge in this field.

## 5 Strategies of surface topology modification

Improvement of the implant’s bioactivation to achieve enhanced interfacial bonding and osseointegration has been classically addressed by various surface engineering methods. These methods mainly focused on increasing the roughness and hydrophilicity by constructing micro, nano or nano/micro hierarchical structure of the implant surface (Wang X. et al., 2016; Wang G. et al., 2019; Sun et al., 2021). The surface roughness is associated with increased wettability. Surface-roughening procedures may also affect the surface chemical composition of the implants, which impacts the hydrophilicity of the implant surface (Junker et al., 2009). A study has shown that micro roughness induces hydrophobicity but subsequently enhances hydrophilicity compared with Ti surfaces without microstructuring (Rupp et al., 2004). Surface modification with high roughness and hydrophilicity were capable of promoting the proliferation and differentiation of cells and achieving higher rates of osseointegration (Wennerberg et al., 2014). The following techniques can be utilized either individually or in combination. Each method has its own specific advantages and limitations (Table 1).

**TABLE 1:** Characteristics of the surface modification techniques for microtopography.

Types	Techniques	Process	Advantages	Limitations
Uncontrolled Microtopography	Plasma Spraying	Thermal spraying technique; Vacuum deposition	Easy to operate; High bonding strength; Economical	Leakage of ions; Difficult to coat inner surface of small holes
	Grit Blasting	Forcing abrasive particles against the implant surface	Simple and low-cost; Roughen the surface	Low processing efficiency; Blasting material residue
	Chemical Etching	Removing materials and fabricating roughness	Low-cost; Leaf-like, needle-like or pyramid-like nanostructures can be obtained	Depending on acid concentration, temperature, and time
	Anodic Oxidation	An accelerated electrochemical process	Simple process; High hardness; Nanometer features; High stability; Enhancing the corrosion resisitance	High energy consumption; Bonding strength with matrix needs to be further improved
Controlled Micro- topography	Laser Treatment	A physical technique of high density form	Able to fabricate complex and high resolution topography; Rapid and clean; Good repeatability	Optimization of all parameters is a big challenge; Multiple treatment sessions and limited
	Photo-lithography	Selectively dissolving photosensitive polymer and leaving latent on substrate	Ideal for microsturcture	Usually requires flat surface and needs chemical post-treatment
	Hot Embossing	Form a relief pattern at an elevated temperature by pressing master into the polymer	Cost-effcctive; Precise; Rapid, and mass production	Restricted to thermoplastics and difficult to fabricate comlex 3D micro- structures
	Micro-milling	Material-removal process using microscale milling tools	Simple; Without affecting the key characteristics of implant surface	Slow and inefficient; Restricted by the available smallest diameter of milling cutters

### 5.1 Uncontrolled microtopography

#### 5.1.1 Plasma spraying

Plasma spray is one of the methods to produce roughness that projected out from the implant surface. This is a thermal spraying technology using plasma arc as heat source and has been widely used in the biomedical field. The technology is simple and easy to operate, and provides a cost-effective, straightforward, and reliable approach for constructing materials with micron scale characteristics on implant surface. Many parameters involved in this method, which can potentially affect the microstructure of coatings. Plasma spray was highly irregular in form, but it can stimulate osteoblast differentiation *in vitro* and support osteogenesis *in vivo* (Ratha et al., 2022; Udduttula et al., 2022). Liu et al. proposed a novel vapor-induced poreforming atmospheric plasma spraying to prepare bioactive porous HA coating with the potential to promote osteoblast attachment and differentiation (Liu et al., 2020b). However, plasma spray implants were gradually replaced by implants with more stable and improved surface morphology in the marketplace because of the leakage of ions, which were taken up by the surrounding cells (Rautray et al., 2011). In addition, the temperature of plasma spray is extremely high, and the coating surface subjects to large thermal stresses. More attention should be paid to the improvement in the preparation of coatings on small and special-shaped workpieces.

#### 5.1.2 Grit blasting

Blasting the implants with forcing abrasive particles against the surface is another approach for roughening the surface., thereby facilitating cell adhesion. According to the size of grit particles and the length of sand blasting process, pits of various sizes can be created on the implant surface (Guehennec et al., 2007). Roughness was in the scale of hundreds of microns to tens of microns. Different ceramic particles have been utilized, such as aluminum oxide, titanium oxide, calcium phosphate and magnesium sulfate particles. Experimental studies have indicated a higher bone-to-implant contact for blasted surfaces in comparison with control surfaces (Hoornaert et al., 2020). Other experiments have demonstrated higher marginal bone levels and survival rates for TiO_2_ grit-blasted implants than for machined turned implants (Steenberghe et al., 2000; Sirello et al., 2018). Grit blasting may also have the disadvantage of low processing efficiency. The blasting material is commonly embedded into the implant surface and residue remains even after ultrasonic cleaning, acidic passivation and sterilization (Guehennec et al., 2007).

#### 5.1.3 Chemical etching

Chemical etching including acid, alkali, hydrothermal treatments and other similar treatment have been reported to develop superhydrophobic surfaces (Kim et al., 2019; Lian et al., 2020). This method consists of a selective and controlled corrosion process and is one of the most promising methods for its commercial application development, due to its low manufacturing costs (Sun et al., 2020). It has been reported that leaf-like, needle-like or pyramid-like nanostructures can be developed on implant surface by autoclaving in presence of distilled water or NaOH solution at 120–240°C (Obata and Kasuga, 2007). The nest-like structure on the surface of metal implants can be prepared by utilizing hydrothermal method in KOH (Wandiyanto et al., 2020) or NaOH solution (Lin et al., 2014), most of which are hydrothermally treated at 110–150°C for 2–24 h, and some need to be calcined at 450–500°C for 2–4 h. However, high KOH/NaOH concentration and longer fabrication duration (often exceeds 24 h) limit the usage of this method. Hydrothermal utilizes aqueous solution as the reaction system in a specific sealed reactor, such as autoclave. Hydrothermal treatment is considered as the inexpensive technique with high engineering potential for irregularly shaped implants (Srivas et al., 2019). Generally, this method often utilizes expensive apparatus and corrosive fluoride based chemicals, has a long reaction cycle, restricts equipment requirements and has technical difficulties such as strict temperature and pressure control.

#### 5.1.4 Anodic oxidation

Anodic oxidation is an accelerated electrochemical process, in which oxide film is applied to the implants surface while immersed in an electrolyte bath. It is now ordinarily utilized to increase the thickness of TiO_2_ layer and fabricate controllable nanostructures on titanium implant surfaces (Liu et al., 2020a). Several possible morphologies can be produced by anodic oxidation technique, which depend not only on cell voltage, but also on the composition of the electrolytic solution, in which the electrodes are immersed. Microarc oxidation (MAO) is developed based on conventional anodizing technology and its process predominantly relies on the matching adjustment of the electrolyte and the electrical parameters. The process is performed on the surfaces of Ti and other valve metals and their alloys at instantaneous high temperatures and pressures generated by arc discharge (Xue et al., 2020). By the principle of plasma electrolytic oxidation, MAO can generate a macro porous and tightly adherent TiO_2_ film on the Ti substrate, which got a lot of attention (Zhang L. et al., 2020; Shen et al., 2020). The enhanced surface hydrophilicity of the porous coating prepared by MAO can stimulate the interaction between the implant and the surrounding biological environment. Although anodic oxidation is convenient and economical, the bonding strength with implant matrix needs to be further improved. The multilevel structure designs, multiscale coating or coating with novel surface morphology should also be developed.

### 5.2 Controlled microtopography

Micromachining may be the best way to prepare an implant surface in a controlled manner. Micro/nano fabrication provides a wide opportunity for the modification of surface with ordered shape, size, and spatial arrangement for controlling or inducing stem cell differentiation. Compared with traditional mechanical and chemical processing methods, it has the advantages of high precision, less pollution, flexible use and high controllability.

#### 5.2.1 Laser treatment

Laser treatment is a contactless process, which does not pollute the implant surface, and has the advantages of wide applicability, high resolution, fast speed, good repeatability (Vanithakumari et al., 2021; Chen et al., 2022). Femtosecond laser micromachining has been efficiently applied to supply microtopography in implant surface (Carvalho et al., 2018; Stanciuc et al., 2018; Wang C. et al., 2019; Trueba et al., 2021). The principle of femtosecond laser is that the laser pulses travelled through air to the focusing device, which can focus the beam and position the sample. The sample is placed on a motorised platform with three-axis motion, so that the pulse can impinge perpendicularly onto its surfaces (Luo et al., 2021). Femtosecond laser technology has the advantages of high precision and low heat production on the material surface (Vanaei et al., 2021). Femtosecond laser treatment can significantly increase the surface roughness, hydrophilicity and surface area of the implant, and reduce residual elements, which provides greater potential for the integration of material and bone (Sun et al., 2021). Many previous studies on the use of laser treatment of ceramic surfaces reported that the hydrophilicity of the surfaces was improved (Hao et al., 2005; Hao and Lawrence, 2006). However, the optimization of all parameters, including power density, scanning speed, frequency and pulse duration, is still a big challenge.

#### 5.2.2 Photolithography

Photolithography is widely used in the microelectronics industry to create micropatterns on flat surfaces. Desired pattern in titanium and silicon have also been generated by photolithography (Kim et al., 2018; Ponomarev et al., 2019). Mainly, the patterning of a layer of photosensitive polymer (photoresist) was produced by utilizing UV or X-ray light at first. The light is shone through a patterned mask giving the designed pattern in the form of UV-opaque features on a UV-transparent background. Then, the desired pattern was transferred to the substrate by dry etching or wet etching of the uncoated areas (Kang et al., 2017). Photolithography is an ideal method for fabricating microstructure, but the disadvantage is that it usually requires a flat surface to start with and needs chemical post-treatment.

#### 5.2.3 Hot embossing

Hot embossing is another technique used for micropatterning. Sun et al. produced microgrooved polystyrene substrates by hot embossing and demonstrated the effect of microgrooved PS surfaces on the morphology, metabolic activity, proliferation and osteogenic differentiation of MG-63 osteoblast-like cells (Sun et al., 2016). Briefly, hot embossing imprint lithography is to press a micromachined master directly into the polymer at an elevated temperature to form a relief pattern. The major advantages of this technology are cost-effectiveness, accuracy and the ability to generate 3D features. However, this technology is limited to thermoplastics and it is difficult to fabricate complex 3D structures (Chen et al., 2022).

#### 5.2.4 Micro-milling

As one of the micromachining methods, micro-milling technology provides high flexibility by manufacturing complex 3D microscale products and has promising applications in preparation of controllable, microscale surface modification, which provides firm support for the surface modification of titanium (Yue et al., 2019; Zhang X. et al., 2020; Kiswanto et al., 2020). By selecting appropriate cutting parameters, superior surface quality can be obtained using micro-milling. The cutting force, temperature, vibration and deflection have a great impact on the quality of the machining process and results. Several studies have been done to identify the appropriate cutting parameters in micro-milling process of Ti6Al4V (Bandapalli et al., 2018; Dadgari et al., 2018). Cai et al. manufactured microstructures on stainless steel using micro-milling, and investigated the effect of microstructure on surface contact angle (Cai et al., 2017).

It should be noted that the aforementioned strategies could be combined to achieve synergies and special structures to improve biological responses (Liang et al., 2022). Generally, microstructures at micron level can be prepared by sandblasting, acid etching, micromachining, electrochemical machining, etc, while nanoscale surface microstructure can be achieved by hydrothermal, alkali heat treatment, anodic oxidation and other technologies. Although all scales of topography are capable of inducing cell alterations, different scales can influence the osteogenic behavior of cells in different ways due to the cellular interaction with the surrounding environment. Nanoscale topography regulates cell behavior primarily through spatial restrictions on the location and size of adhesion complexes, which can modulate cell adhesion to adsorbed proteins through integrin and consequently induce intracellular expression of target genes (Davison et al., 2016). Cell-matrix interaction with microscale topography generates effects by modifying the entire cell morphology, that is, inducing cell deformation due to cytoskeletal remodeling to adapt to substrate shape (Rabel et al., 2020). Natural bone is a hierarchical structure containing macroscale, microscale, and nanoscale organizations (Wang X. et al., 2016). From a biomimetic perspective, micro/nano textured surface can mimic the structure of bone tissue and enhance cell responses. The micro/nano hierarchical architecture has been accounted to accelerate cell functions by synergistic equilibrating between cellular proliferation and differentiation (Srivas et al., 2019; Sun et al., 2022). Studies have shown that it is possible to create hierarchical textures through the combination of micro and nano features on the same surface. For instance, Maher et al. (Maher et al., 2021) created bioinspired multistructured surfaces on SLM-printed Ti6Al4V implants by combining electrochemical anodization and hydrothermal methods. The implants display unique surfaces with a distinctive dual micro to nano topography composed of micron-sized spherical features and vertically aligned nanoscale pillar structures, which can increase the deposition of hydroxyapatite minerals in simulated body fluids and the adhesion of human osteoblast-like cells. It should be pointed out that most of the modification strategies resulted in a surface that had a complex multi-scale roughness. The hydrophilic properties added to the roughened surfaces have shown higher biocompatibility and have induced faster osseointegration, compared to the existing modified surfaces (Yeo, 2014). After roughening the surface by combination of sandblasting and acid etching (SLA), increased hydrophilicity can promote bone formation and exhibit higher osteogenic potential compared to samples without modifications to hydrophilic properties (Wall et al., 2009). A hydrophilization technique evaluated clinically used phosphonic acid coupled to blasted and acid-etched surfaces to generate a “biomimetic” surface with increased hydrophilicity (Esposito et al., 2013). Taken together, it is essential to choose an appropriate method in terms of implant materials, applying situations and fabricating procedures. Construction of hierarchical microtopography mimicking native bone on the implant surface is an effective modification strategy to improve cell responses and osseointegration.

## 6 Summary

For a long-term and stable fixation of implants, osseointegration has been considered as a pivotal process. Osseointegration is affected by various factors including materials, surface microtopography, the environment of bone-implant interface, the design of the implant itself, and so on. Materials determine the principal characteristics of implants. Qualified materials should have enough mechanical properties, high corrosion and good biocompatibility (Liu et al., 2020b). Surface modification is used to modify the chemical, physical, and biological properties of implant surface for better osseointegration and has raised increasing attention in modern orthopedic medicine recently. Moreover, bone is a 3D inhomogeneous structure with complex topography. Porous implants that have a similar hierarchical structure on multiple scales with native bone may facilitate osseointegration. The pores enhance the permeability of implants and create the space for nutrient exchanges, which grant better biocompatibility and osseointegration potential for implants (Cheng et al., 2018).

Microtopography of the implant surface is potent modulator of the osseointegration. Different cell types prefer various microenvironments. Fibroblasts adhere more strongly to smooth surfaces while rough surfaces facilitate the adhesion and proliferation of osteoblasts. Modifying the biomaterial surface properties to enhance host cell adhesion and function has been a major focus. Biomaterial surfaces with physical properties of roughness or hydrophilicity and have complex HMN surface structure, are more effective in supporting osseointegration. Roughness, hydrophilicity and chemical composition are the bridges connecting the physicochemical properties and biological properties of implant surfaces. Although it is possible to induce stem cells differentiation towards the osteoblast lineage by controlling the microtopography, no optimal micropattern structure and size have been confirmed yet. To further analyze the connection between cell behaviors and the microtopography, potential mechanotransduction mechanisms need to be investigated. At present, Wnt/β-catenin and YAP/TAZ signal pathways were recognized as mediating the regulation of cell adhesion and cytoskeleton organization on the microtopography, which ultimately affected the expression of osteogenic genes.

A large number of surface treatment approaches emerged for modifying implant surfaces with the purpose of increasing surface roughness. These modification techniques can be combined to create hierarchical textures mimicking host bone structure. However, each method has its own specific advantages and limitations. It is still urgent to develop a simple, efficient, easy-to-operate, safe, and controllable method or to combine various surface modification methods to play a synergistic effect, and combine their advantages to conquer deficiencies. Nonetheless, it is increasingly accepted that infection is also a major cause of implant failure. Implant surfaces that support osseointegration may also favor colonization of bacterial cells. Therefore, to improve the success of implants, biomaterial surfaces should ideally discourage the attachment of bacteria without affecting osteogenic cell functions (Spriano et al., 2018). Future strategies should explore a combined goal.

In conclusion, surface microtopography of the implant plays a very important role in osseointegration. Further research is still required on the preparation of tailored and standardized microtopography. In addition, how cells sense the microenvironment and transform mechanical cues into intracellular signals, the underlying mechanisms of interaction between microtopography and cells need further investigation, which could help to create tailored implant surfaces to promote bone formation around the implant.
